# Prevalence of eunatremic, eukalemic hypoadrenocorticism in dogs with signs of chronic gastrointestinal disease and risk of misdiagnosis after previous glucocorticoid administration

**DOI:** 10.1111/jvim.16921

**Published:** 2023-12-06

**Authors:** Antonio Maria Tardo, Francesca Del Baldo, Rodolfo Oliveira Leal, Giorgia Galiazzo, Marco Pietra, Alba Gaspardo, Federico Fracassi

**Affiliations:** ^1^ Department of Veterinary Medical Sciences University of Bologna, Ozzano dell'Emilia Bologna Italy; ^2^ CIISA – Centre for Interdisciplinary Research in Animal Health, Faculty of Veterinary Medicine University of Lisbon Lisbon Portugal; ^3^ Associate Laboratory for Animal and Veterinary Sciences (AL4AnimalS) Lisbon Portugal

**Keywords:** Addison's disease, atypical hypoadrenocorticism, canine, chronic enteropathy, cortisol

## Abstract

**Background:**

Dogs with eunatremic, eukalemic hypoadrenocorticism (EEH) typically show signs of chronic gastrointestinal disease (CGD). Previous glucocorticoid administration (PGA) can give false‐positive results on the ACTH stimulation test (ACTHst).

**Hypothesis/Objectives:**

To determine the prevalence of EEH in dogs with signs of CGD, and to identify clinical and clinicopathological features for EEH and PGA.

**Animals:**

One hundred twelve dogs with CGD (101 non‐PGA and 11 PGA), 20 dogs with EEH.

**Methods:**

Multicenter prospective cohort study. Basal serum cortisol (BSC) concentration was measured in dogs with signs of CGD. When BSC was <2 μg/dL and in PGA dogs, ACTHst plus measurement of endogenous ACTH (eACTH) were performed. Records of dogs with EEH from 2009 to 2021 were reviewed.

**Results:**

The BSC concentration was <2 μg/dL in 48/101 (47.5%) non‐PGA and in 9/11 (82%) PGA dogs. EEH was diagnosed in 1/112 dog (prevalence 0.9%; 95% CI, 0.1%‐4.8%); the ACTHst provided false‐positive results in 2/11 PGA dogs. PGA dogs showed lower C‐reactive protein‐to‐haptoglobin ratio (median 0.01, range 0.003‐0.08; *P* = .01), and higher haptoglobin (140, 26‐285 mg/dL; *P* = .002) than non‐PGA dogs (0.04, 0.007‐1.5; 38.5, 1‐246 mg/dL, respectively). eACTH was higher (*P* = .03) in EEH (396, 5‐>1250 pg/mL) than in non‐PGA dogs (13.5, 7.3‐46.6 pg/mL). Cortisol‐to‐ACTH ratio was lower (*P* < .0001 and *P* = .01, respectively) in EEH (0.002, 0.0002‐0.2) than in non‐PGA (0.1, 0.02‐0.2) and PGA dogs (0.1, 0.02‐0.2).

**Conclusions and Clinical Importance:**

The prevalence of EEH in dogs with signs of CGD was lower than previously reported. The clinical and clinicopathological features herein identified could increase the index of suspicion for EEH or PGA in dogs with an unclear history of glucocorticoid administration.

**Abbreviations**
ACTHst: ACTH stimulation test
BSC: basal serum cortisol
CAR: cortisol‐to‐ACTH ratio
CGD: chronic gastrointestinal disease
CHR: C‐reactive protein‐to‐haptoglobin ratio
CI: confidence interval
eACTH: endogenous ACTH concentration
EEH: eunatremic eukalemic hypoadrenocorticism
HA: hypoadrenocorticism
HPA: hypothalamic‐pituitary‐adrenal
PGA: previous glucocorticoid administration
PU/PD: polyuria and polydipsia
RDW: red cell distribution width
SD: standard deviation
UCCR: urine cortisol‐to‐creatinine ratio

## 1 INTRODUCTION

Hypoadrenocorticism (HA) is a rare endocrinopathy in dogs.^1^ Primary HA refers to bilateral adrenal gland destruction and accounts for more than 95% of cases.^2^ Secondary HA, a much rarer condition, is because of reduced ACTH secretion from the pituitary gland.^2^ In the majority of cases of primary HA, both glucocorticoid and mineralocorticoid secretions are impaired, resulting in hypocortisolemia and electrolyte abnormalities; nevertheless, up to 30% of dogs with primary HA have normal electrolyte concentrations at diagnosis.^3^, ^4^, ^5^ This form of the disease is therefore defined as eunatremic, eukalemic hypoadrenocorticism (EEH), also defined as “atypical” HA.^6^ Dogs with EEH are characterized by a condition of permanent hypocortisolemia usually associated with a low to undetectable aldosterone concentration, despite having normal electrolyte concentrations.^7^, ^8^ Eunatremic, eukalemic hypoadrenocorticism might go undetected for a long period because of vague clinical signs and the absence of typical biochemical abnormalities. Consequently, EEH might be mistaken for other diseases, such as chronic gastrointestinal disease (CGD). Diagnosing HA depends on adrenal gland function testing, such as the ACTH‐stimulation test (ACTHst). However, there have recently been problems with this test, including its high cost and the intermittent availability of exogenous ACTH in some countries.^9^, ^10^ As a result, basal serum cortisol (BSC) concentration, using a cut‐off value of ≥2 μg/dL (>55 nmol/L), is commonly used as a screening test to rule out HA. BSC concentration <2 μg/dL (<55 nmol/L) has excellent sensitivity for HA (99.4%‐100%).^11^, ^12^, ^13^ However, because of the low specificity of the test (20%‐78.2%), up to 33% of dogs with CGD, but without HA, have a BSC <2 μg/dL (<55 nmol/L).^11^, ^12^, ^13^, ^14^, ^15^, ^16^ For this reason, urine cortisol‐to‐creatinine ratio (UCCR) and cortisol‐to‐ACTH ratio (CAR) have been proposed as alternative screening tests for HA in dogs.^14^, ^17^, ^18^, ^19^

Even though the ACTHst remains the gold standard for HA diagnosis, previous glucocorticoid administration (PGA), commonly used in dogs with signs of CGD, can give false positive results. In previous studies, PGA was excluded based only on the history of the dog which likely led to an overdiagnosis of EEH.^3^, ^4^, ^5^, ^15^ Demonstrating a high endogenous ACTH concentration (eACTH) could be an objective method for differentiating EEH from false positive results of the ACTHst because of PGA. Dogs with secondary HA have low eACTH; however, this is a rare condition with marginal clinical relevance.^2^ To confirm the diagnosis of EEH, it is appropriate to consider the anamnesis and the result of ACTHst; however, at the same time, it is important to demonstrate that eACTH is elevated. This study aimed to determine the prevalence of EEH in dogs with signs of CGD and to identify the clinical and clinicopathological features which might help in differentiating dogs with “atypical” HA from those with CGD, and to recognize PGA in dogs with CGD.

## 2 MATERIALS AND METHODS

### 2.1 Study design

A multicenter prospective cohort study involving client‐owned dogs with chronic (>3 weeks) signs routinely seen in dogs with HA, such as vomiting, diarrhea, decreased appetite, weakness or lethargy, from 2 different veterinary hospitals (Veterinary Teaching Hospital of the University of Bologna, Veterinary Teaching Hospital of the University of Lisbon) from June 2019 to December 2021 was carried out. The presence of at least 1 of vomiting or diarrhea was a mandatory inclusion criterion. All the dogs were enrolled according to the study protocol which was approved by the Scientific Ethics Committee of the University of Bologna (no. 1255/2021). In addition, because of the low prevalence of EEH in the dogs with signs of CGD, the medical records of all the dogs with a diagnosis of EEH admitted to the Veterinary Teaching Hospital of the University of Bologna between January 2009 and December 2021 were reviewed.

### 2.2 Animals

The data obtained at the time of enrollment included signalment, history (including previous administration of glucocorticoids), physical examination findings, and laboratory test results which included CBC, serum chemistry profile and urinalysis. The diagnostic workup included measurement of the BSC, and determination of the folate and cobalamin concentrations. When the BSC was <2 μg/dL and in the dogs with PGA, an ACTHst plus the measurement of eACTH were carried out. Fecal flotation and standard egg count were performed in all the dogs if recent fecal testing results were not available. The decision regarding additional diagnostics was the responsibility of the clinician managing the case.

The dogs were divided into 2 groups: those which had not received glucocorticoids in the previous 90 days (non‐PGA) and those which received systemic or topical glucocorticoids which had been suspended for fewer than 90 days before admission (PGA). In the PGA dogs, the ACTHst and the measurement of eACTH were repeated if the post‐ACTH serum cortisol concentration was <3 μg/dL (<83 nmol/L). The time elapsed for repeating the tests was dictated by the dog's clinical signs or the owner's willingness to return for the tests.

A diagnosis of EEH was made if the following criteria were met: (a) post‐ACTH serum cortisol concentration <2.0 μg/dL (<55.0 nmol/L); (b) high (>58 pg/mL) or undetectable (<5 pg/mL) plasma eACTH concentrations, and (c) the absence of electrolyte abnormalities. Dogs with undetectable eACTH were excluded from the EEH group if a glucocorticoid medication had been administered within 90 days before testing.

### 2.3 Endocrine testing and analytical procedures

For the ACTHst, blood samples were taken before and 60 minutes after the IV injection of 5 μg/kg synthetic ACTH (Synacthen, Alfasigma S.P.A., Bologna, Italy). Blood samples for the determination of eACTH concentrations were collected before the injection of synthetic ACTH. The BSC and eACTH concentrations were used for calculating the CAR.

All the analytical procedures were carried out at the veterinary laboratory of the University of Bologna. The samples from Lisbon were stored at −80°C and shipped overnight on dry ice to the veterinary laboratory of the University of Bologna. Blood samples for the determination of the eACTH were collected into EDTA‐coated plastic tubes placed on ice. The samples were immediately centrifuged at 4°C, 500 *g* for 8 minutes, and the plasma was immediately transferred to plastic tubes, stored at 4°C and analyzed within 8 hours, or stored at −80°C and thawed immediately before analysis. Blood samples for the determination of the cortisol were collected in serum separating tubes. Coagulated blood samples were centrifuged for 10 minutes at 3000 *g*; the serum was immediately transferred to plastic tubes, stored at 4°C and analyzed the same day, or stored at −80°C and thawed immediately before analysis. The serum cortisol and eACTH concentrations were measured using a chemiluminescent enzyme immunoassay (Immulite 2000, Siemens Healthcare) which had been validated for dogs and is widely used in laboratories throughout the world.^20^, ^21^

### 2.4 Statistical analysis

Statistical analysis was carried out using commercial statistical software packages (GraphPad Prism 7, San Diego, California). Descriptive statistics were generated to characterize the study population. Continuous variables were presented as median and range (minimum and maximum value). The categorical variables were described with frequencies, proportions or percentages. The overall prevalence of EEH and its 95% confidence interval (CI) according to Wilson were calculated. The differences between the groups (non‐PGA, PGA, and EEH) regarding the categorical and numerical variables were analyzed using the Fisher's exact test and the Kruskal‐Wallis test with Dunn's post‐test, respectively. For dogs with cortisol values reported as “<1 μg/dL,” 0.5 μg/dL was used for statistical calculations. For ACTH values reported as “<5 pg/mL” and “>1250 pg/mL,” 5 and 1250 pg/mL were used for calculations, respectively. The level of significance was set at *P* < .05.

## 3 RESULTS

### 3.1 Animals

A total of 112 dogs were enrolled, including 101 non‐PGA and 11 PGA dogs. One dog was diagnosed with EEH, giving a prevalence estimate of “atypical” HA in this cohort of dogs of 0.9% (95% CI, 0.1%‐4.8%). Sixty‐nine dogs were male, of which 41 were neutered, and 43 were female, of which 22 were spayed. The median age was 3.0 years (range, 6 months to 12.1 years) and the median body weight was 21.9 kg (range, 2.2‐53.5 kg). Mixed breeds (n = 28) were most common, followed by Labrador Retrievers (n = 7), German Shepherds (n = 7), Jack Russell Terriers (n = 7), Weimaraners (n = 5), French Bulldogs (n = 4), and 54 other purebred dogs of 33 different breeds. The most common clinical signs on presentation are reported in Table 1. Polyuria and polydipsia (PU/PD) were more commonly reported in the PGA than the non‐PGA dogs (*P* = .01).

**TABLE 1:** Descriptive statistics of clinical signs in dogs with signs of chronic gastrointestinal disease which had not received glucocorticoids (non‐PGA), dogs with previous glucocorticoid administration (PGA), and dogs with eunatremic, eukalemic hypoadrenocorticism (EEH).

Variable	Non‐PGA	PGA	EEH
	n	n	n
	%	%	%
Vomiting	69 69	9 81	14 70
Diarrhea	83 83	11 100	16 80
Weakness or lethargy	12^a^ 12	4 36	12^a^ 60
Decreased appetite	29 ^a^ 29	6 55	13^a^ 65
Anorexia	13 13	1 9	0 ‐
Weight loss	30 30	3 27	2 10
Hematochezia	26 26	1 9	2 10
Melena	5 5	0 ‐	0 ‐
Polyuria/polydipsia	3^a^ 3	3^a,b^ 27	0^b^ ‐

*Note*: Data are presented as frequencies and percentages. Significant differences (*P* < .05) between groups are shown with the same superscript symbols (a‐b).

In the PGA dogs, prednisolone was the most commonly used medication in 8/11, followed by prednisone and topical triamcinolone (otic and cutaneous routes), and betamethasone (ophthalmic route), and hydrocortisone aceponate (cutaneous route) in 1 dog each. The median dose was 0.5 mg/kg (range, 0.14‐0.9). The median time of glucocorticoid treatment and discontinuation was 60 (range, 2‐300) and 25 (range, 6‐63) days, respectively.

#### 3.1.1 Dogs with EEH

Eunatremic, eukalemic hypoadrenocorticism was the final diagnosis in 1 dog, a 7‐year‐old female spayed Miniature Pinscher with a 6‐month history of weight loss, vomiting, diarrhea, and sporadic episodes (2‐3 times a month) of hematemesis and hematochezia.

In addition, data from 19 dogs with EEH were retrospectively collected. Ten dogs were male, of which 3 were neutered, and 9 were female, all of which were spayed. Eleven different breeds were counted. The most represented breeds were mixed breed (n = 6) and Jack Russell Terrier (3), followed by 1 each of Golden Retriever, Pekingese, Samoyed, Siberian Husky, Boxer, Labrador Retriever, Ibizan Hound, German Shepherd, Pomeranian, and Border Collie. The median age of the dogs with EEH was 5.4 years (range, 1.3‐11.7 years) and the median body weight was 19 kg (range, 3.7‐36.2 kg). The clinical signs are reported in Table 1. Decreased appetite (*P* = .003) and weakness or lethargy (*P* < .0001) were more commonly reported in EEH dogs when compared with non‐PGA dogs whereas PU/PD were more common in PGA than in EEH dogs (*P* = .04).

Endogenous ACTH concentration was available in 16/19 (84%) dogs. Based on eACTH concentration, primary and secondary EEH was diagnosed in 11/16 (69%) and 5/16 (31%) dogs, respectively. Endogenous ACTH measurement was not available in the remaining 3 dogs. All the dogs diagnosed with EEH were treated with glucocorticoids and did not receive mineralocorticoid replacement. One or more concurrent diseases were documented in 12/19 (63%) dogs, including 7 (37%) with primary inflammatory enteropathy (6/7 food‐responsive enteropathy and 1/7 immunosuppressant‐responsive enteropathy); 2 (10%) with hypothyroidism; and 1 each with diabetes mellitus, immune‐mediated thrombocytopenia, lymphoma, or cutaneous mast cell tumors. Follow‐up information was available in 12/19 (63%) dogs with a median follow‐up time of 284 days (range, 31‐2201). Of these, none of the dogs developed electrolytes abnormalities after diagnosis.

### 3.2 Laboratory findings and adrenal testing

The laboratory variables of the non‐PGA, PGA, and EEH dogs are shown in Table 2. Dogs with EEH showed significantly lower hematocrit (*P* = .0005), serum albumin concentration (*P* = .0001) and albumin‐to‐globulin ratio (*P* = .007), and higher red cell distribution width (RDW, *P* = .01) than non‐PGA dogs. Moreover, the EEH dogs had lower serum albumin (*P* = .02), and higher potassium (*P* = .03) concentrations when compared with the PGA dogs. Haptoglobin concentration was significantly higher (*P* = .002) and the C‐reactive protein‐to‐haptoglobin ratio (CHR) significantly lower (*P* = .01) in the PGA than in the non‐PGA dogs.

**TABLE 2:** Results of clinicopathological variables in dogs with signs of chronic gastrointestinal disease which had not received glucocorticoids (non‐PGA), dogs with previous glucocorticoid administration (PGA), and dogs with eunatremic, eukalemic hypoadrenocorticism (EEH).

Variable (unit)	Non‐PGA		PGA		EEH		*P* value
	Result (n)	Range	Result (n)	Range	Result (n)	Range	
Hematocrit (%)	50.5 (98)^a^	27.4‐62	46.8 (11)	42.2‐56.2	43.5 (20)^a^	34.4‐55.8	**.0006**
MCV (fL)	69.1 (98)	59.7‐80	70.2 (11)	65.2‐78.3	68.9 (20)	62.8‐76.9	.51
MCHC (g%)	34.1 (98)	31.9‐37.2	34.5 (11)	32.1‐36	33.6 (20)	30.8‐36.4	.08
RDW (%)	12.1 (96)^a^	10.3‐20.8	12.4 (11)	11.3‐12.8	13.4 (20)^a^	10.6‐17.7	**.01**
WBC (×10^3^/μL)	9.7 (98)	3.3‐45.2	9.9 (11)	5.8‐14.5	8.9 (20)	5.1‐34.6	.79
Neutrophils (×10^3^/μL)	5.8 (98)	1.5‐29.4	6.5 (11)	3.7‐9.5	5.1 (20)	3.2‐29.1	.63
Lymphocytes (×10^3^/μL)	2.2 (98)	0.6‐6.7	1.8 (11)	0.5‐3.4	2.4 (20)	1‐4.6	.10
Monocytes (×10^3^/μL)	0.48 (98)	0.08‐2.6	0.32 (11)	0.23‐1.1	0.44 (20)	0.15‐1.5	.23
Eosinophils (×10^3^/μL)	0.43 (97)	0‐2.3	0.18 (11)	0.07‐1.7	0.35 (20)	0.06‐1.9	.58
Platelets (×10^3^/μL)	252 (98)	51‐600	269 (11)	142‐436	274 (20)	4‐521	.39
Glucose (mg/dL)	95 (97)	62‐153	95 (11)	81‐114	91 (19)	50‐115	.63
Fructosamine (μmol/L)	244 (74)	128‐396	226 (9)	171‐479	225 (13)	203‐347	.79
ALT (U/L)	46 (98)	23‐518	50 (11)	22‐858	60.5 (20)	12‐315	.22
AST (U/L)	38 (89)	12‐89	32 (11)	18‐66	40 (19)	13‐146	.43
ALP (U/L)	39.5 (96)	11‐368	51 (11)	16‐309	56 (20)	12‐300	.34
GGT (U/L)	3.1 (87)	0‐9.4	2.3 (10)	0.3‐18.8	3.2 (19)	0.8‐18.8	.70
Total bilirubin (mg/dL)	0.21 (83)	0.06‐0.31	0.18 (11)	0.09‐0.35	0.2 (20)	0.07‐03	.79
Total proteins (g/dL)	6.3 (96)	4.3‐7.5	6.3 (11)	5.3‐7.2	6.1 (20)	5‐6.9	.05
Albumin (g/dL)	3.2 (98)^a^	1.5‐3.9	3.3 (11)^b^	2.5‐3.9	2.9 (20)^a,b^	1.6‐3.6	**.001**
Albumin/globulin ratio	1.07 (96)^a^	0.6‐3.3	1.1 (11)	0.7‐1.2	0.9 (20)^a^	0.5‐1.5	**.007**
Cholesterol (mg/dL)	186.5 (88)	75‐403	213 (11)	134‐821	174 (20)	54‐381	.2
Triglycerides (mg/dL)	45.5 (76)	22‐161	50 (10)	27‐260	67 (15)	23‐144	.69
Urea (mg/dL)	36 (96)	17‐125	38 (11)	29‐89	39 (20)	19‐131	.25
Creatinine (mg/dL)	1.05 (98)	0.6‐1.7	1 (11)	0.5‐1.3	0.9 (20)	0.6‐1.6	.83
CRP (mg/dL)	1.1 (77)	0.7‐26.3	1.1 (9)	0.8‐6.1	1.5 (12)	0.6‐10.2	.4
Haptoglobin (mg/dL)	38.5 (72)^a^	1‐246	140 (9)^a^	26‐285	69 (10)	12‐212	**.002**
CRP/Haptoglobin ratio	0.04 (72)^a^	0.007‐1.5	0.01 (9)^a^	0.003‐0.08	0.05 (10)	0.004‐2.8	**.01**
Calcium (mg/dL)	10.2 (84)	8.2‐11.3	10.3 (11)	9.6‐11.4	9.9 (20)	9.3‐10.6	.05
Phosphate (mg/dL)	4 (84)	1.6‐8.5	4 (11)	3.4‐5.9	4.1 (20)	2.7‐6.2	.43
Sodium (mEq/L)	148 (98)	130‐159	148	141‐157	148 (20)	140‐153	.39
Potassium (mEq/L)	4.4 (98)	3.7‐5.3	4.1 (11)^a^	3.6‐5.1	4.8 (20)^a^	3.6‐5.3	**.03**
Chloride (mEq/L)	113.4 (91)	90.8‐124	111 (11)	102‐122	113.7 (20)	103.7‐120	.14
Cobalamin (mEq/L)	436.5 (88)	150‐1000	371.5 (6)	219‐584	509 (8)	216‐1118	.58
Folate (μg/dL)	6.3 (88)	1‐24	7.2 (6)	6.5‐15.4	9.7 (8)	1‐18.2	.23
Urinary specific gravity	1042 (49)	1006‐1080	1044 (8)	1030‐1062	1042 (16)	1029‐1070	.94
UPC	0.13 (26)	0.06‐1.7	0.3 (6)	0.1‐2.6	0.1 (15)	0.07‐0.6	.13

*Note*: Data are presented as median and range (min‐max value). The *P*‐values in bold correspond to statistically significant findings (*P* < .05). Significant differences between groups are shown with the same superscript symbols (a‐b).

Abbreviations: ALP, alkaline phosphatase; ALT, alanine aminotransferase; AST, aspartate transaminase; CRP, C‐reactive protein; GGT, gamma‐glutamyl transferase; MCHC, mean corpuscular hemoglobin concentration; MCV, mean corpuscular volume; RDW, red cell distribution width; UPC, urine protein‐to‐creatinine ratio; WBC; white blood cells.

**FIGURE 1:**
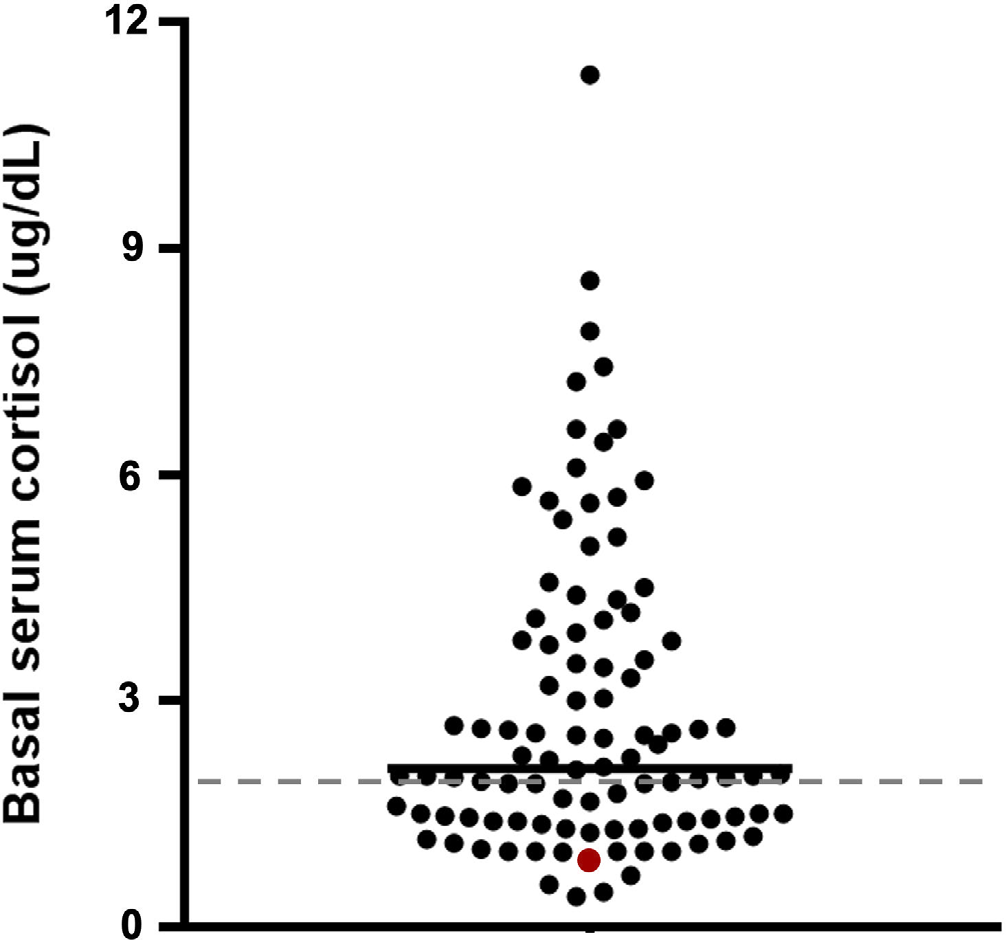
Basal serum cortisol concentration in 101 dogs with signs of chronic gastrointestinal disease which had not received glucocorticoids. The dashed gray line represents a 2 μg/dL (55 nmol/L) cut‐off for excluding hypoadrenocorticism. The red dot represents the dog with the final diagnosis of eunatremic, eukalemic hypoadrenocorticism.

**FIGURE 2:**
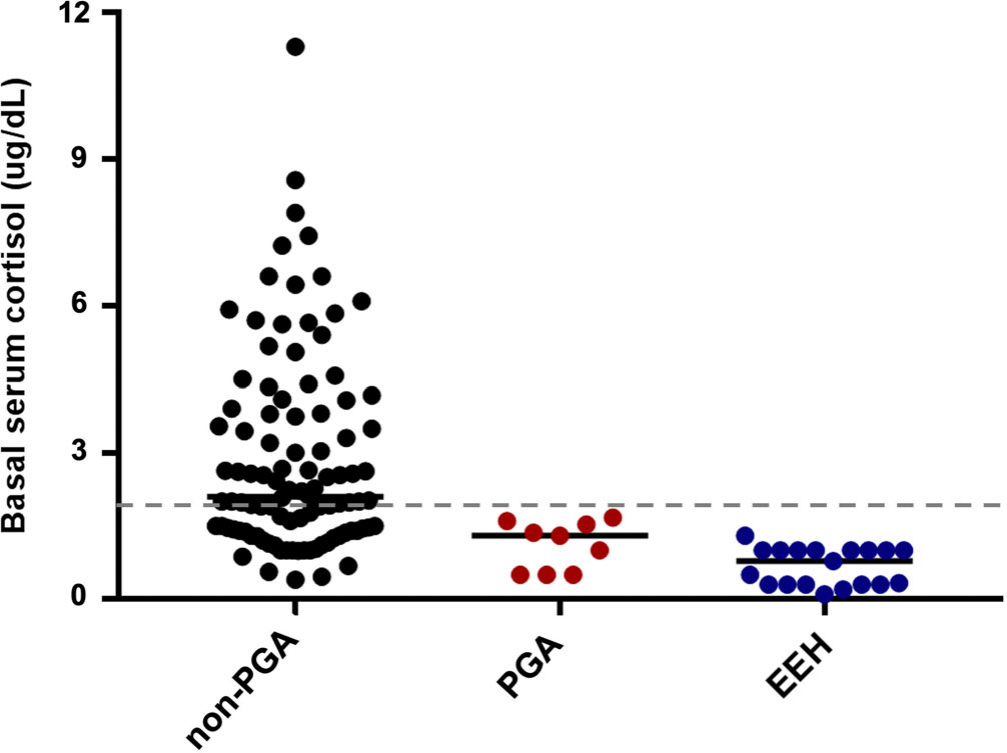
Comparison of basal serum cortisol concentration in dogs with signs of chronic gastrointestinal disease which had not received glucocorticoids (non‐PGA) (n = 100), dogs with previous glucocorticoid administration (PGA) (n = 9), and dogs with eunatremic eukalemic hypoadrenocorticism (EEH) (n = 20). The dashed gray line represents a 2 μg/dL (55 nmol/L) cut‐off for excluding hypoadrenocorticism. The horizontal bars represent the median values.

**FIGURE 3:**
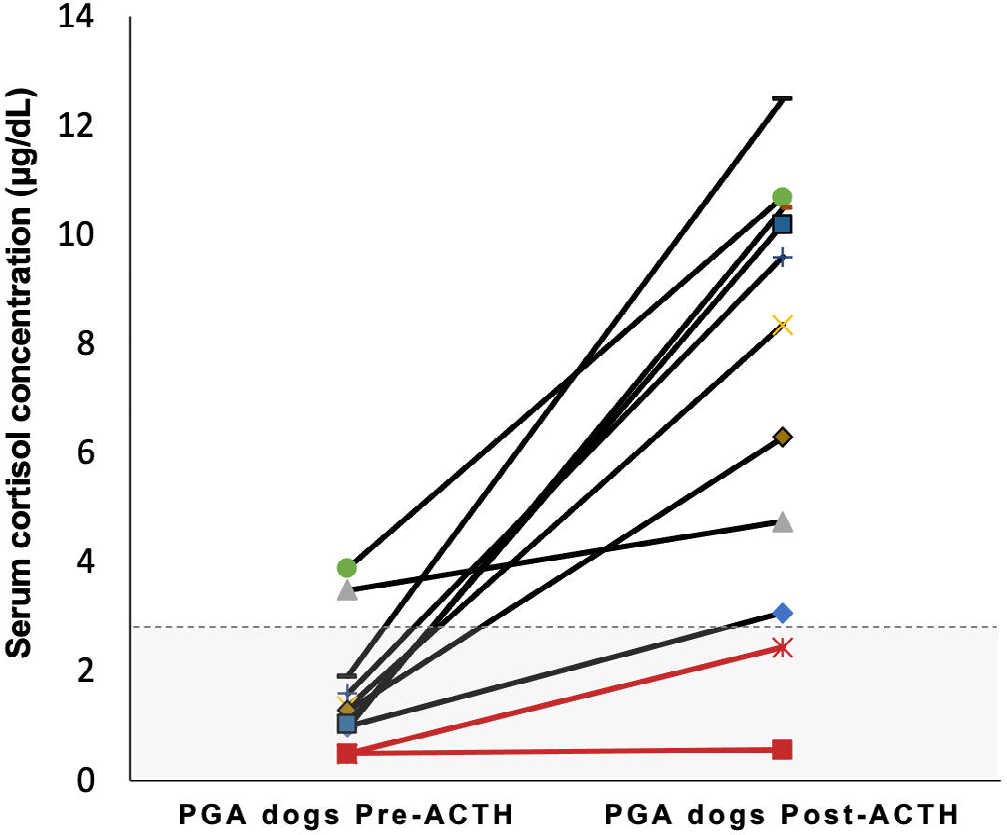
Serum cortisol concentrations before and after stimulation with synthetic ACTH in 11 dogs with previous glucocorticoid administration (PGA). The dashed gray line represents a 3 μg/dL (83 nmol/L) cut‐off.

**FIGURE 4:**
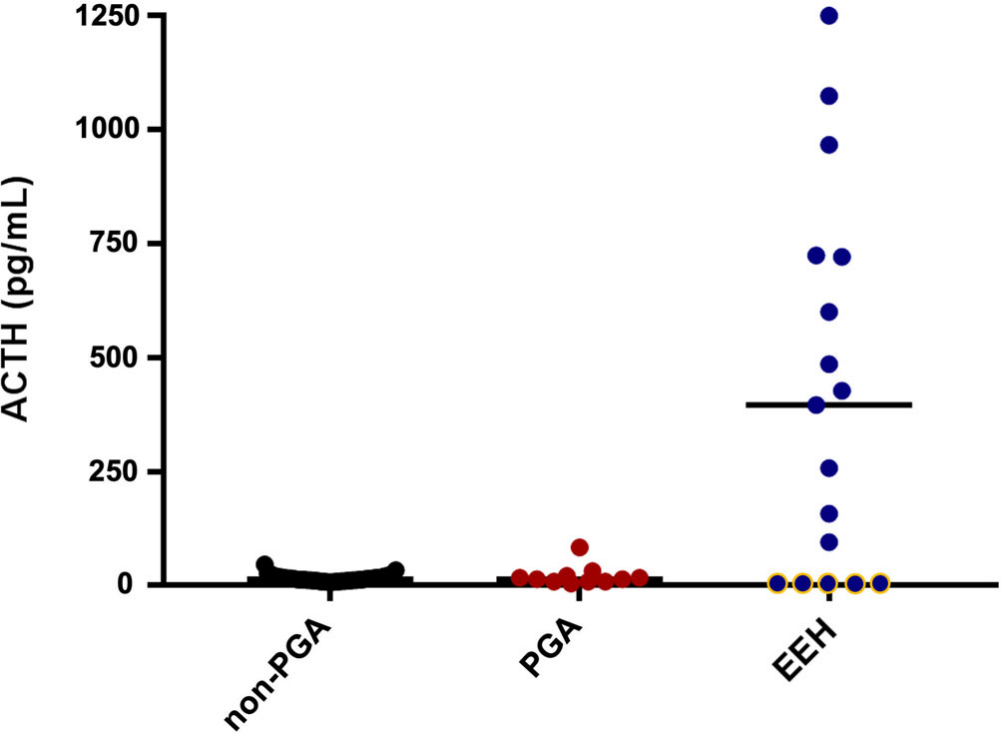
Comparison of the endogenous ACTH (eACTH) concentration in dogs with signs of chronic gastrointestinal disease which had not received glucocorticoids (non‐PGA) (n = 100), dogs with previous glucocorticoid administration (PGA) (n = 11), and dogs with eunatremic eukalemic hypoadrenocorticism (EEH) (n = 20). The dots with yellow borders represent dogs with secondary hypoadrenocorticism. The horizontal bars represent the median values.

**FIGURE 5:**
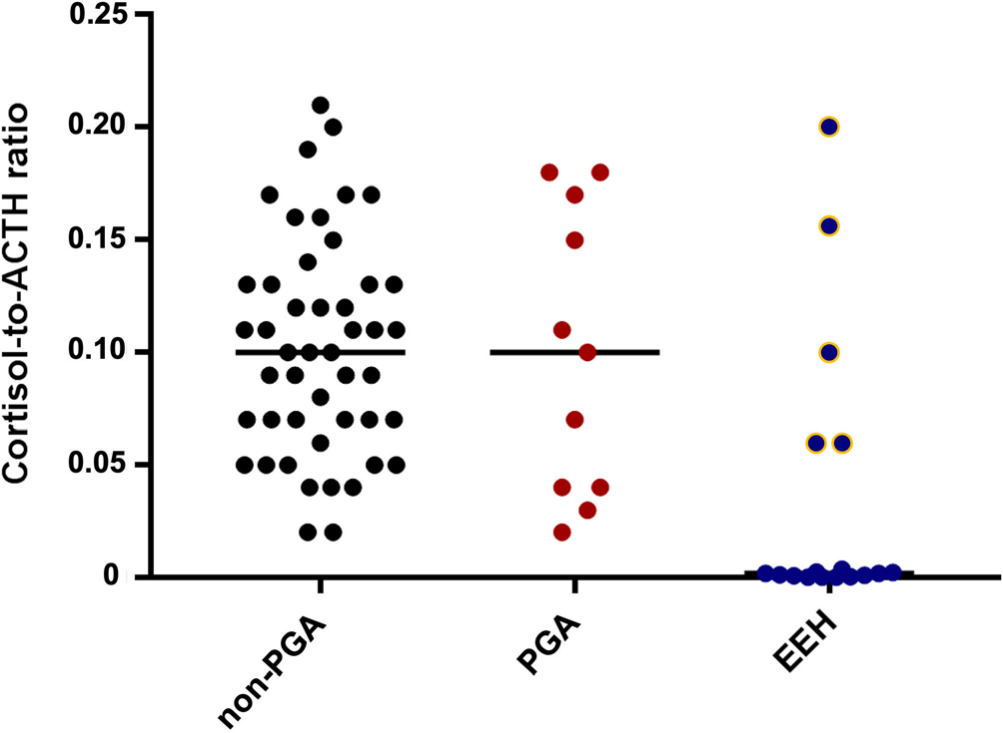
Comparison of the cortisol‐to‐ACTH ratio (CAR) in dogs with signs of chronic gastrointestinal diseases which had not received glucocorticoids (non‐PGA) (n = 100), dogs with previous glucocorticoid administration (PGA) (n = 11), and dogs with eunatremic eukalemic hypoadrenocorticism (EEH) (n = 20). The dots with yellow borders represent dogs with secondary hypoadrenocorticism. The horizontal bars represent the median values.

The adrenal test results are reported in Table 3. In the non‐PGA dogs, 48/101 (47.5%) had BSC <2 μg/dL (Figure 1), and only 1 dog was diagnosed with EEH (post‐ACTH cortisol 1.51 μg/dL; eACTH > 1250 pg/mL). The BSC was <2 μg/dL in all the PGA dogs in which it was measured (9/11, 82%) and was significantly lower (*P* = .008) when compared with the non‐PGA dogs (Figure 2). The ACTHst provided a false‐positive result in 2/11 PGA dogs (Figure 3); the first dog had been treated with 0.3 mg/kg prednisolone for 7 months for suspected EEH and, at the time of admission, the glucocorticoids had been discontinued for 6 days; a second dog had been treated for 30 days before the ACTHst with 0.9 mg/kg prednisolone for 4 days. In these dogs, the eACTH was low‐normal (5 and 17.5 pg/mL, respectively) and the repeated ACTHst (after 14 and 33 days, respectively) was normal. The dogs with EEH showed lower BSC (*P* < .0001) and post‐ACTH cortisol (*P* < .0001), and higher eACTH (*P* = .03; Figure 4) than the non‐PGA dogs. Moreover, the CAR was significantly lower (*P* < .0001 and *P* = .01, respectively) in the EEH dogs when compared with the non‐PGA and the PGA dogs (Figure 5).

**TABLE 3:** Results of adrenal testing in dogs with signs of chronic gastrointestinal disease which had not received glucocorticoids (non‐PGA), dogs with previous glucocorticoid administration (PGA), and dogs with eunatremic, eukalemic hypoadrenocorticism (EEH).

Variable (unit)	Non‐PGA		PGA		EEH		*P* value
	Result (n)	Range	Result (n)	Range	Result (n)	Range	
Basal cortisol (μg/dL)	2.1 (100)^a,b^	0.4‐11.3	1.3 (9)^a^	0.5‐1.7	0.6 (20)^b^	0.1‐1.3	**<.0001**
Post‐ACTH cortisol (μg/dL)	9.9 (47)^a^	3.2‐16.8	8.3 (11)^b^	0.6‐12.5	1 (20)^a,b^	0.2‐1.9	**<.0001**
Endogenous ACTH (pg/mL)	13.5 (47)^a^	7.3‐46.6	14.2 (11)	5‐83.4	396 (17)^a^	5‐>1250	**.03**
Cortisol‐to‐ACTH ratio	0.1 (47)^a^	0.02‐0.2	0.1 (9)^b^	0.02‐0.2	0.002 (17)^a,b^	0.0002‐0.2	**.0001**

*Note*: Data are presented as median and range (min‐max value). The *P*‐values in bold correspond to statistically significant findings (*P* < .05). Significant differences between groups are shown with the same superscript symbols (a‐b).

## 4 DISCUSSION

In this multicenter prospective study, the prevalence of EEH in a cohort of dogs with CGD presented to 2 referral institutions was 0.9% (95% CI, 0.1%‐4.8%). The estimated prevalence in the present study closely corresponded with the overall prevalence of HA in the general canine population (between 0.06% and 1.1%),^1^, ^22^, ^23^, ^24^ and with that recently reported in a large group of dogs with signs of CGD.^16^ However, the results of this study demonstrated a lower prevalence of EEH than previously described in dogs with signs of CGD presented to several referral centers in Germany and in the Netherlands.^15^ In the latter study, 6 of the 151 (4%) dogs with signs of CGD were diagnosed with HA and none of these dogs had abnormalities in serum electrolyte concentrations.^15^ Unfortunately, as in many other studies,^5^, ^15^, ^16^, ^26^ eACTH was not measured which might have led to an overestimation of the true prevalence of EEH. Moreover, it remains unknown whether those cases suffered from primary HA or if some of them might have had secondary HA. It is important to remember that the measurement of eACTH remains a cornerstone for the diagnosis of HA, especially when abnormalities in serum electrolyte concentrations are not detected.^2^ Failure to evaluate this variable might result in a distorted prevalence of EEH. In addition, the differences between the 2 studies could have been because of the different inclusion criteria, especially with regard to PGA. In the present study, the ACTHst provided a false positive result in 2 dogs with PGA. It should be noted that 1 of the dogs had been treated with prednisolone for only 4 days and eACTH was found to be undetectable 30 days after glucocorticoid discontinuation. Based on these findings, the dog could have been diagnosed as having secondary HA. It is therefore important to exclude PGA in dogs with low pre‐ and post‐ACTH cortisol concentrations. Glucocorticoids lead to suppression of the hypothalamic‐pituitary‐adrenal (HPA) axis and its duration varies based on preparation, dose, and individual sensitivity to steroid drugs.^25^ To date, there are no published guidelines regarding the delay required for adequate HPA recovery after the administration of systemic or topical glucocorticoids. Due to the marked inter‐individual variability, in the authors' opinion, it is difficult to establish the best timing for carrying out an ACTHst after glucocorticoids discontinuation. Even in this context, eACTH measurement appears to be of utmost importance as it allows the clinician to suspect iatrogenic HA.

The present study identified clinical and laboratory variables that were significantly different between the PGA dogs and the other study groups. Polyuria and polydipsia were more commonly reported in the PGA dogs when compared with the EEH and the non‐PGA dogs. Glucocorticoids have the potential of causing PU/PD; this might result in an increased perception of these signs by the owner. Hence, PU/PD should raise the clinician's index of suspicion for PGA. However, PU/PD can also be caused by gastrointestinal diseases and should be interpreted carefully in a dog with signs of CGD.^27^ Serum albumin and potassium concentrations were significantly different between the PGA and the EEH dogs; however, substantial overlap was observed. In the current study, serum haptoglobin concentration was significantly higher and the CHR significantly lower in the PGA than in the non‐PGA dogs. Haptoglobin is a moderate acute‐phase protein particularly sensitive to glucocorticoids; elevated concentrations are found both after treatment with glucocorticoids and during naturally occurring hypercortisolism.^28^, ^29^ However, haptoglobin is a positive acute‐phase protein and results could be biased if a dog has a concomitant inflammatory disease such as CGD. In this context, measurement of the CHR could be useful for differentiating PGA from non‐PGA dogs since exogenous glucocorticoid treatment appears to blunt the magnitude of C‐reactive protein elevation in dogs with inflammatory state.^29^ The finding of high serum haptoglobin concentration and low CHR in a dog with signs of CGD should alert the clinician to consider the possibility of PGA. Given the present results, these measurements should be considered to be a part of the diagnostic work‐up when PGA is suspected.

In the current study, 47.5% of the non‐PGA and all the PGA dogs had a BSC concentration <2 μg/dL (<55 nmoL/L). This finding was consistent with other investigations, demonstrating that up to 33% of dogs with nonadrenal illness have a BSC concentration <2 μg/dL (<55 nmoL/L).^11^, ^12^, ^13^, ^14^, ^15^, ^16^ Moreover, BSC was significantly lower in the PGA than in the non‐PGA dogs. Determination of the BSC concentration has a high sensitivity (100% if <2 μg/dL) for HA, but a low specificity of only 20%‐78.2%.^11^, ^12^, ^13^, ^14^ Therefore, because of the low specificity of the test, an ACTHst should be carried out in dogs with BSC <2 μg/dL (<55 nmol/L) in order to exclude HA. In this study, an ACTHst was carried out in approximately half of the dogs with signs of CGD which are the signs most commonly screened for HA in clinical practice, with a consequent increase in diagnostic cost and time for the client. This raises the question of whether BSC concentration should be measured in all dogs with signs of CGD. For this reason, the CAR and the UCCR have been proposed as alternative screening tests for HA in dogs.^14^, ^17^, ^18^, ^19^ The CAR is a valuable and reliable tool for diagnosing primary HA, the advantage of which is that only a single blood sample needed.^14^, ^17^ However, the diagnostic utility of this test can be limited in clinical practice owing to the critical sampling collection and handling needed for eACTH measurement. The present results were in agreement with previous reports^14^, ^17^ and demonstrated that the CAR could be considered a useful diagnostic test for discriminating EEH dogs from those with signs of CGD and PGA. However, when also considering dogs with secondary HA some overlap between the groups was detected. In dogs with secondary HA, the UCCR could be more useful as compared to the CAR. Recent studies have shown the excellent diagnostic performance of the UCCR in dogs with HA, having a reported sensitivity and specificity ranging from 97.2% to 100% and 93.6% to 97.3%, respectively.^18^, ^19^ The anti‐cortisol antibody used in the chemiluminescent enzyme immunoassay has recently been changed by the manufacturer, which indicates that the UCCR performance reported herein might need to be validated again with a new assay. Since the authors did not evaluate the UCCR, it is not possible to draw any conclusion regarding this test.

The present study described a large cohort of dogs with EEH in which eACTH was measured. Based on eACTH concentration, primary and secondary EEH was diagnosed in 69% and 31% of dogs, respectively. Secondary HA because of the reduced secretion of eACTH from the pituitary gland is considered a rare cause of adrenocortical failure (fewer than 5% of cases),^2^ and its prevalence in dogs with EEH has not been reported in any study. Dogs with EEH were more likely to have decreased appetite, weakness or lethargy when compared with non‐PGA dogs. These differences reflected the fact that “atypical” cases might go undetected for a longer period since the clinical signs are non‐specific and often wax and wane. However, data from EEH dogs were retrospectively collected, and these cases were selected based on their diagnosis rather than signs of CGD. This might have biased the comparison between groups with regard to clinical signs. Concurrent diseases were documented in 63% of the dogs, and the majority of the comorbidities were immune disorders. Immune‐mediated destruction of the adrenal gland in humans is commonly associated with other immune disorders.^2^ Polyglandular autoimmune disease is rare in dogs, with HA and hypothyroidism being the most common concurrent disorders.^2^ Hypothyroidism was diagnosed in 2/19 (10%) dogs with EEH, and the most common comorbidity was primary inflammatory enteropathy. However, it is difficult to determine whether signs of gastrointestinal disease in dogs with EEH might be related to disruptions of the epithelial barrier of the gastrointestinal tract because of cortisol deficiency or concurrent primary gastrointestinal disease. A small percentage of dogs with EEH can develop abnormalities in serum electrolyte concentrations indicative of mineralocorticoid deficiency weeks to months after diagnosis.^2^, ^3^, ^7^ Interestingly, none of the EEH dogs developed electrolyte abnormalities over a follow‐up time of 31 days to 6 years, but aldosterone concentrations were not measured. Therefore, one cannot be sure that the dogs enrolled did not have some degree of mineralocorticoid deficiency.

The present study identified clinicopathologic features, including hematocrit, RDW, serum albumin, and an albumin/globulin ratio which were significantly different between the EEH and the non‐PGA dogs. These variables could be utilized to increase the index of suspicion for EEH; however, substantial overlap was observed, indicating that gold standard adrenal function testing should be carried out in dogs with a compatible clinical presentation regardless of clinicopathological abnormalities.

The present study had some limitations, including the small sample size in the EEH and the PGA groups which could have influenced the statistical power. Data from the dogs with EEH were collected retrospectively, and the absence of some parameters might have partially biased the results. This study was not designed to investigate the HPA recovery time. Hence, the ACTHst was carried out at different times after glucocorticoid discontinuation and was not carried out at a specific predetermined time. Furthermore, the glucocorticoid dose was different for each dog in the PGA group. This might have influenced the results; however, it reflected the real condition of the clinical setting.

In conclusion, the prevalence of EEH in dogs with signs of CGD was lower than previously reported. The results of this study showed that glucocorticoid administration, even for a few days, could cause false positive results on the ACTHst. The clinical and clinicopathological variables identified in the present study could increase the index of suspicion for EEH or PGA in dogs with an unclear history of glucocorticoid administration. Since dogs with HA require lifelong treatment, it is important to measure eACTH and to repeat the ACTHst when PGA is suspected.

## CONFLICT OF INTEREST DECLARATION

Authors declare no conflict of interest.

## OFF‐LABEL ANTIMICROBIAL DECLARATION

Authors declare no off‐label use of antimicrobials.

## INSTITUTIONAL ANIMAL CARE AND USE COMMITTEE (IACUC) OR OTHER APPROVAL DECLARATION

Approved by the Scientific Ethics Committee of the University of Bologna (number 1255/2021).

## HUMAN ETHICS APPROVAL DECLARATION

Authors declare human ethics approval was not needed for this study.

## References

[1] Hanson JM , Tengvall K , Bonnett BN , Hedhammar Å . Naturally occurring adrenocortical insufficiency—an epidemiological study based on a swedish‐insured dog population of 525,028 dogs. J Vet Intern Med. 2016;30:76‐84. 26683136 10.1111/jvim.13815 PMC4913634

[2] Scott‐Moncrieff JC . Hypoadrenocorticism. In: Feldman EC , Nelson RW , Reusch CE , Scott‐Moncrieff JC , Behrend E , eds. Canine and Feline Endocrinology. 4th ed. St. Louis: Elsevier; 2015:213‐257.

[3] Thompson AL , Scott‐Moncrieff JC , Anderson JD . Comparison of classic hypoadrenocorticism with glucocorticoid‐deficient hypoadrenocorticism in dogs: 46 cases (1985–2005). J Am Vet Med Assoc. 2007;230:1190‐1194. 17501661 10.2460/javma.230.8.1190

[4] Adamantos S , Boag A . Total and ionised calcium concentrations in dogs with hypoadrenocorticism. Vet Rec. 2008;163:25‐26. 18603632 10.1136/vr.163.1.25

[5] Kelly D , Garland M , Lamb V , et al. Prevalence of ‘Atypical’ Addison's disease among a population of dogs diagnosed with hypoadrenocorticism. (Abstract ESVE O‐2). ECVIM‐CA Congress, 19‐21 September 2019, Milan – Italy.

[6] Rogers W , Straus J , Chew D . Atypical hypoadrenocorticism in three dogs. J Am Vet Med Assoc. 1981;179:155‐158. 6267000

[7] Baumstark ME , Sieber‐Ruckstuhl NS , Müller C , Wenger M , Boretti FS , Reusch CE . Evaluation of aldosterone concentrations in dogs with hypoadrenocorticism. J Vet Intern Med. 2014;28:154‐159. 24428320 10.1111/jvim.12243 PMC4895548

[8] Cartwright JA , Stone J , Rick M , Dunning MD . Polyglandular endocrinopathy type II (Schmidt's syndrome) in a Dobermann pinscher. J Small Anim Pract. 2016;57:491‐494. 27487017 10.1111/jsap.12535

[9] Peterson ME . Containing cost of ACTH‐stimulation test. J Am Vet Med Assoc. 2004;224:198‐199. 14736059

[10] Kemppainen RJ , Behrend EN , Busch KA . Use of compounded adrenocorticotropic hormone (ACTH) for adrenal function testing in dogs. J Am Anim Hosp Assoc. 2005;41:368‐372. 16267060 10.5326/0410368

[11] Lennon EM , Boyle TE , Hutchins RG , et al. Use of basal serum or plasma cortisol concentrations to rule out a diagnosis of hypoadrenocorticism in dogs: 123 cases (2000–2005). J Am Vet Med Assoc. 2007;231:413‐416. 17669044 10.2460/javma.231.3.413

[12] Bovens C , Tennant K , Reeve J , Murphy KF . Basal serum cortisol concentration as a screening test for hypoadrenocorticism in dogs. J Vet Intern Med. 2014;28:1541‐1545. 25066405 10.1111/jvim.12415 PMC4895569

[13] Gold AJ , Langlois DK , Refsal KR . Evaluation of basal serum or plasma cortisol concentrations for the diagnosis of hypoadrenocorticism in dogs. J Vet Intern Med. 2016;30:1798‐1805. 27714859 10.1111/jvim.14589 PMC5115184

[14] Boretti FS , Meyer F , Burkhardt WA , et al. Evaluation of the cortisol‐to‐ACTH ratio in dogs with hypoadrenocorticism, dogs with diseases mimicking hypoadrenocorticism and in healthy dogs. J Vet Intern Med. 2015;29:1335‐1341. 26250121 10.1111/jvim.13593 PMC4858040

[15] Hauck C , Schmitz SS , Burgener IA , et al. Prevalence and characterization of hypoadrenocorticism in dogs with signs of chronic gastrointestinal disease: a multicenter study. J Vet Intern Med. 2020;34:1399‐1405. 32573832 10.1111/jvim.15752 PMC7379021

[16] Gallego AF , Gow AG , Boag AM . Evaluation of resting cortisol concentration testing in dogs with chronic gastrointestinal signs. J Vet Intern Med. 2022;36:525‐531. 35118742 10.1111/jvim.16365 PMC8965248

[17] Lathan P , Scott‐Moncrieff JC , Wills RW . Use of the cortisol‐to‐ACTH ratio for diagnosis of primary hypoadrenocorticism in dogs. J Vet Intern Med. 2014;28:1546‐1550. 24966067 10.1111/jvim.12392 PMC4895572

[18] Del Baldo F , Gerou Ferriani M , Bertazzolo W , Luciani M , Tardo AM , Fracassi F . Urinary cortisol‐creatinine ratio in dogs with hypoadrenocorticism. J Vet Intern Med. 2022;36:482‐487. 35150029 10.1111/jvim.16358 PMC8965274

[19] Moya MV , Refsal KR , Langlois DK . Investigation of the urine cortisol to creatinine ratio for the diagnosis of hypoadrenocorticism in dogs. J Am Vet Med Assoc. 2022;260:1041‐1047. 35417417 10.2460/javma.21.12.0538

[20] Singh AK , Jiang Y , White T , Spassova D . Validation of nonradioactive chemiluminescent immunoassay methods for the analysis of thyroxine and cortisol in blood samples obtained from dogs, cats, and horses. J Vet Diagn Invest. 1997;9:261‐268. 9249165 10.1177/104063879700900307

[21] Scott‐Moncrieff JC , Koshko MA , Brown JA , Hill K , Refsal KR . Validation of a chemiluminescent enzyme immunometric assay for plasma adrenocorticotropic hormone in the dog. Vet Clin Pathol. 2003;32:180‐187. 14655102 10.1111/j.1939-165x.2003.tb00333.x

[22] Bellumori TP , Famula TR , Bannasch DL , Belanger JM , Oberbauer AM . Prevalence of inherited disorders among mixed‐breed and purebred dogs: 27,254 cases (1995‐2010). J Am Vet Med Assoc. 2013;242:1549‐1555. 23683021 10.2460/javma.242.11.1549

[23] Kelch WJ . Canine Hypoadrenocorticism (Canine Addison's Disease): History, Contemporary Diagnosis by Practicing Veterinarians, and Epidemiology [PhD dissertation]. Knoxville: University of Tennessee; 1996.

[24] Schofield I , Woolhead V , Johnson A , Brodbelt DC , Church DB , O'Neill DG . Hypoadrenocorticism in dogs under UK primary veterinary care: frequency, clinical approaches and risk factors. J Small Anim Pract. 2021;1–8:343‐350. 10.1111/jsap.13285 PMC8248152 33555046

[25] Reusch CE . Glucocorticoid therapy. In: Feldman EC , Nelson RW , Reusch CE , Scott‐Moncrieff JC , Behrend E , eds. Canine and Feline Endocrinology. 4th ed. St. Louis: Elsevier; 2015:555‐577.

[26] Reagan KL , McLarty E , Marks SL , Sebastian J , McGill J , Gilor C . Characterization of clinicopathologic and abdominal ultrasound findings in dogs with glucocorticoid deficient hypoadrenocorticism. J Vet Intern Med. 2022;36:1947‐1957. 36326216 10.1111/jvim.16564 PMC9708419

[27] Polzin DJ . Polyuria and polydipsia. In: Washabau RJ , Day MJ , eds. Canine and Feline Gastroenterology. 1st ed. St. Louis: Elsevier; 2012:151‐156.

[28] Martinez‐Subiela S , Ginel PJ , Ceròn JJ . Effects of different glucocorticoid treatments on serum acute phase proteins in dogs. Vet Rec. 2004;154:814‐817. 15260442 10.1136/vr.154.26.814

[29] McGrotty Y , Bell R , McLauchlan G . Disorders of plasma proteins. In: Villiers E , Ristic J , eds. BSAVA Manual of Canine and Feline Clinical Pathology. 3rd ed. England: BSAVA; 2016:123‐141.

